# Genome Sequence of a Brazilian Bovine Enterovirus

**DOI:** 10.1128/mra.01200-21

**Published:** 2022-02-10

**Authors:** Ana C. S. Mosena, Mariana S. da Silva, Juliana S. Gularte, Meriane Demoliner, Viviane Girardi, Eduardo F. Flores, Rudi Weiblen, Fernando R. Spilki

**Affiliations:** a Laboratório de Microbiologia Molecular, Universidade Feevale, Novo Hamburgo, Rio Grande do Sul, Brazil; b Setor de Virologia, Departamento de Medicina Veterinária Preventiva, Universidade Federal de Santa Maria, Santa Maria, Rio Grande do Sul, Brazil; KU Leuven

## Abstract

We report the nearly complete genome sequence of a Brazilian bovine enterovirus (genus *Enterovirus*, family *Picornavirus*). This enterovirus was isolated from an enteric and respiratory disease outbreak in a beef cattle herd in southern Brazil. Phylogeny indicates that this isolate belongs to the species *Enterovirus E*.

## ANNOUNCEMENT

Bovine enteroviruses (BEVs), belonging to the species *Enterovirus E* and *Enterovirus F* (1), may be associated with enteric and respiratory disease in cattle (2). Enteroviruses have a 7,100- to 7,450-nucleotide-long RNA genome, containing a single open reading frame (ORF). BEVs are endemic in cattle worldwide but, despite its large commercial cattle herd, data about BEV infection in Brazil are scarce. After a serological screening performed in the 1970s (3), the presence of these viruses determined through reverse transcriptase (RT)-PCR was described only in 2016 (4). Since then, no other data on BEV in Brazil have been published. Here, we report the nearly complete genome sequence of a Brazilian BEV.

The UFSM-SV89/91 virus was isolated from an outbreak of respiratory and intestinal signs in a beef cattle herd in Rio Grande do Sul state, Brazil, in 1991. Calves presented nasal vesicles and ulcers. Virus isolation was achieved in primary bovine cells using nasal secretions and turbinate homogenates. Cytopathic effect compatible with picornaviruses was observed. An inoculum was preserved in liquid nitrogen but, due to technical limitations at that time, there were no successful attempts to further characterize the isolate at the molecular level. Recently, total RNA from MDBK cell culture supernatants inoculated with UFSM-SV89/91 was extracted using a PureLink RNA kit, and cDNA was obtained with SuperScript IV reverse transcriptase using random primers (Invitrogen). cDNA was submitted for high-throughput sequencing (HTS) using the Illumina MiSeq platform after library preparation using the Nextera XT kit (2 × 150-bp paired-end reads), resulting in a total of 106,584 reads. *De novo* assembly using SPAdes v3.6 yielded the complete genome sequence of an enterovirus. Default parameters were used for all software unless specified.

**FIG 1:**
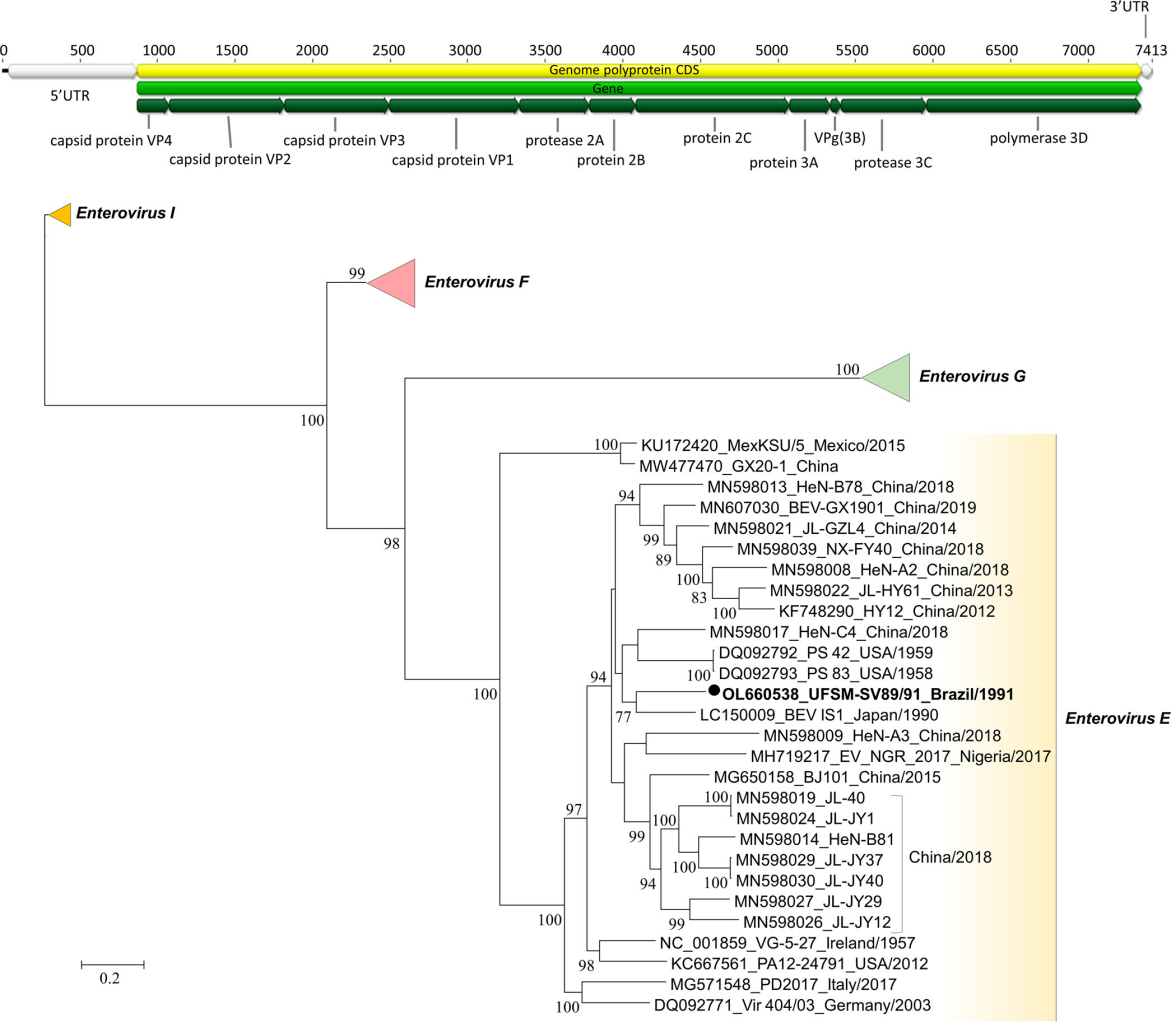
Genomic architecture and phylogenetic analysis of the SV89/91 BEV. (Top) Schematic diagram of the genomic organization of the UFSM-SV89/91 *Enterovirus E* isolate. UTR, untranslated region; CDS, coding sequence. (Bottom) Phylogenetic tree based on the nucleotide sequence of the complete genome, reconstructed using the maximum likelihood method. One thousand bootstrap replicates were used, and the percentages of replicates with the same clustering position are shown next to the branches (values of <70% were omitted). Evolutionary distances were computed using Smart Model Selection (SMS) in PhyML v3.0 and the Akaike information criterion (AIC). Sequences used in the phylogeny were retrieved from GenBank, and accession numbers for the *Enterovirus E* clade are shown, as well as the isolate name, country, and year of collection. Sequences representing the other species clades (*Enterovirus F*, *Enterovirus G*, and *Enterovirus I* member species) were collapsed. The UFSM-SV89/91 sequence is shown in bold and indicated with a black circle.

The UFSM-SV89/91 genomic contig obtained was 7,370 nucleotides long and composed of 93,305 reads. ORF annotation was obtained through the map-to-reference tool in Geneious Prime v10.2.2 software (reference GenBank accession number NC_001859) and confirmed using a BLASTx approach. A typical enterovirus genome structure was obtained (Fig. 1, top). The mean genome coverage per site was approximately 700×, and the GC content was 50.1%. The genome was found to be 4 nucleotides shorter at the 3′ end, compared to the reference genome. The UFSM-SV89/91 sequence was subjected to BLASTn analysis, and it was found to be closely related to that of an *Enterovirus E* strain from Japan (IS1/Bos taurus/JPN/1990 [GenBank accession number LC150009]) that was isolated in the 1990s, with a nucleotide identity of 81.14%.

The classification of species within the *Enterovirus* genus is performed through phylogeny; therefore, bovine (*Enterovirus E* and *Enterovirus F*), swine (*Enterovirus G*), and dromedary (*Enterovirus I*) enterovirus complete genomes were selected from GenBank and aligned using the MUSCLE algorithm in MEGA v6.06, and the maximum likelihood phylogeny was determined with the online tool PhyML v3.0 (Fig. 1, bottom). UFSM-SV89/91 clustered with *Enterovirus E* genomes, and it is related to the aforementioned Japanese isolate (Fig. 1, bottom).

In a previous report of BEV identification in Brazil (4), the strains were classified as *Enterovirus F* species. However, the sequences analyzed were 180 nucleotides long and were not considered for inclusion in this phylogenetic analysis. The addition of more ancient and recent Brazilian genomes in the future will improve the understanding of the phylogenetic relationships. Our finding confirms previous sparse results on BEV circulation in Brazil and represents an early step to elucidate the enteroviruses circulating among Brazilian cattle.

### Data availability.

The complete genome sequence of the UFSM-SV89/91 isolate has been deposited in GenBank under the accession number OL660538. Raw reads are available in the SRA under the accession number PRJNA791934.
